# Treatment mode and prognosis of esophageal perforation after radiotherapy in patients with esophageal carcinoma

**DOI:** 10.3389/fonc.2022.961902

**Published:** 2023-01-12

**Authors:** Zhen Chan-Jun, Bai Wen-Wen, Zhang Ping, Song Yu-Zhi, Wang Ya-Jing, Qiao Xue-Ying, Zhou Zhi-Guo

**Affiliations:** Department of Radiation Oncology, The Fourth Hospital of Hebei Medical University, Hebei, Shijiazhuang, China

**Keywords:** esophageal carcinoma, radiotherapy, esophageal perforation, prognosis, treatment

## Abstract

**Objective:**

Retrospectively analyzed the esophageal carcinoma (EC) patients with esophageal perforation (EP) after radiotherapy to discuss the treatment and prognosis.

**Methods:**

Data of patients with EC who had EP after radiotherapy in Hebei Cancer Hospital were collected from 2001 to 2020 and retrospectively analyzed. All analyses were performed using SPSS Statistics for Windows, version 18. 0 (SPSS Inc., Chicago, Ill., USA). *P* values less than 0.05 were considered statistically significant.

**Results:**

A total of 94 patients with EC were enrolled, among which 72 were males and 22 were females, with a median age of 62 (38–82) years. The tumor was located in the upper thoracic in 45 patients, middle thoracic in 45 patients, and lower thoracic in 4 patients. There were 30 cases of tracheoesophageal fistula (TEF) and 64 cases of esophagomediastinal fistula (EMF). All patients died within 11 months (median: two months) after EP. After EP, 48 patients were treated by tube feeding (include nasal feeding and gastrostomy), 26 patients by esophageal stenting, and 20 patients by fluid infusion therapy, and their one, three, and six months survival rates after EP were 81.3%, 31.3%, and 12.5% (P = 0.000). In the TEF group, the one, three, and six month survival rates after EP of tube feeding, esophageal stenting and fluid infusion groups were 88.2%, 17.6%, 11.8%; 45.5%, 27.3%, 0%; and 50.0%, 50.0%, 0% (*P* = 0.345). In the EMF group, the one, three, and six months survival rates after EP of this three groups were 77.4%, 38.7%, 12.9%; 26.7%, 20.0%, 6.7%; and 22.2%, 11.1%, 0% (*P*=0.002), respectively.

**Conclusion:**

Most patients with EP after radiotherapy died within six months, with low survival and poor prognosis. Tube feeding therapy can achieve relatively good survival, especially for patients with EMF. The survival of patients treated by tube feeding therapy is significantly better than the survival of those treated by other methods.

## Introduction

In China, there is a high incidence of esophageal carcinoma (EC) (in which approximately 90% of the cases are referred to as esophageal squamous cell carcinoma) (1). Chemoradiotherapy (CRT) is the standard method of treatment and good curative effects have been achieved for unresectable EC (2–6). However, CRT may damage the wall of the esophagus, resulting in malnutrition, as well as imbalance between tumor shrinkage and normal tissue repair, eventually leading to esophageal perforation (EP) (7–9). Anatomically, the thoracic esophagus is adjacent to large vessels, pericardium, trachea, bronchus, vertebrae, and other tissues. At the advanced stage of EC, the normal structure of the above-mentioned normal tissues is subject to invasion and destruction, which leads to the formation of EP. EP is a severe clinical complication that can occur during or after radiotherapy in patients with EC. Although the incidence of EF is low (10.4%-13.9%) but the prognosis is poor (10, 11). T4 stage, N3 stage, re-RT, ulcerative EC, esophageal stenosis, the maximum thickness of the tumor might be the risk factors for EP (12–14). Once EP occurs, oral feeding is immediately stopped and substituted with stents, nutrition tubes or gastrojejunostomy to prevent further contamination of the peri-fistula tissues by esophageal contents. Treatment options for patients with EC complicated with EP include RT, CRT, surgery, stent placement, and conservative treatment, but there is no consensus. Moreover, there are few studies reporting the treatment and prognosis of EP after radiotherapy in patients with EC. Therefore, this study aimed to retrospectively analyze the clinical data of a total of 94 patients with EC who had EP after radiotherapy in our hospital from June 2001 to January 2020. Herein, we discuss the treatment mode of EP that occurred after radiotherapy in patients with EC to provide clinical treatment options for similar patients.

## Materials and methods

### Inclusion criteria

Data of patients with EC who had EP after radiotherapy in Hebei Cancer Hospital were collected from February 2001 to 2020 and retrospectively analyzed. The inclusion criteria were as follows: 1.) pathologically confirmed esophageal squamous cancer, 2.) non-surgical treatment, such as treatment by radiotherapy with or without chemotherapy, 3.) no sign of EP before radiotherapy, 4.) confirmed EP after radiotherapy; 5.) without serious medical illness, with a KPS score > 70, and 6.) with no other types of cancer, except EC.

### Definition of EP

The common clinical manifestations of EP include dramatic cough with massive sputum or hematemesis, chest pain and fever. Once the patients were suspected of having EP, enhanced computed tomography (CT) scan and/or barium esophagography were performed. Barium esophagography could continuously and dynamically monitor the contrast agent passing through the perforation and show the location, size and shape of perforation. The findings of mediastinal fluid and mediastinal air on CT are strongly suggestive of EP. EP including esophagomediastinal fistula (EMF) and tracheoesophageal fistula (TEF).

### Data analysis

Data were summarized and analyzed retrospectively. Overall survival (OS) time was defined as the time from the beginning of radiotherapy to the last follow-up or death. The survival time after esophageal perforation (TAP) in this study was defined as the time from the diagnosis of esophageal perforation to the last follow-up or death. *P* values less than 0.05 were considered statistically significant. All analyses were performed using SPSS Statistics for Windows, version 18. 0 (SPSS Inc., Chicago, Ill., USA).

## Results

### Clinical data of patients

A total of 94 patients with EC were enrolled, among which 72 were males and 22 were females, with a median age of 62 (38–82) years. Of the 94 patients with EC, the tumor was located in the upper thoracic in 45 patients, middle thoracic in 45 patients, and lower thoracic in 4 patients. The length of the esophageal tumor lesions was in the range of 2.5–15.0 cm, with a median length of 6.0 cm. All the patients were treated with radiotherapy at the dosage of 20–68 Gy, with a median dose of 60 Gy. Eighteen patients were treated with concurrent chemoradiotherapy (CRT), while 22 patients were treated with sequential CRT. However, most of the applications were TP, DP, and FP. There were 30 cases of TEF and 64 cases of EMF. Seventeen cases of the EP occurred during radiotherapy, while 77 cases occurred after radiotherapy with a median interval between RT and EP of 6.5 (2.0–26.0) months. The patient characteristics are listed in **Table 1**.

**Table 1:** Patients characteristics.

		No.(%)
Gender	Male	72(77.1)
	Female	22(22.9)
Age (years)	<60	38(45.9)
	≥60	56(54.1)
Location of primary tumor	Upper thoracic	45(55.7)
	Mid thoracic	45(41.0)
	Lower thoracic	4(3.3)
Total radiation dose (Gy)	<60	28(26.2)
	≥60	66(73.8)
Concurrent CRT	Yes	18(18.0)
	No	76(82.0)
Sequential CRT	Yes No	24(25.5) 70(74.5)
Type of EP	TEF	30(29.5)
	EMF	64(70.5)
Treatment of EP	Gastrostomy	17(14.8)
	Nasal feeding	31(26.2)
	Stening	26(32.8)
	Fluid infusion	20(26.2)

### Therapeutic method

After EP, 31 patients were treated by nasal feeding, 17 patients were treated by gastrostomy, 26 patients were treated by esophageal stenting, and 20 patients were treated by fluid infusion therapy.

### Survival

**Figure 1:**
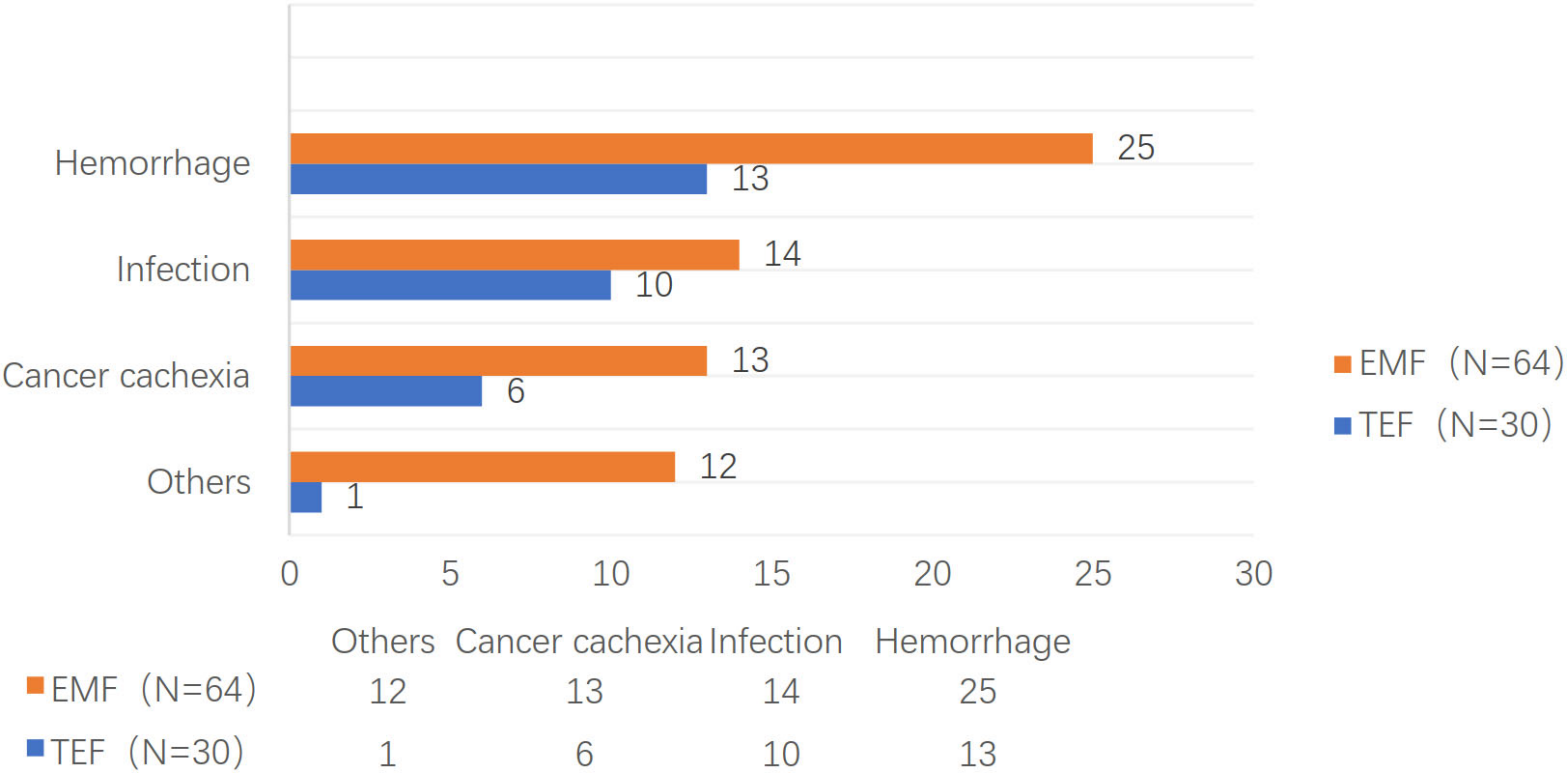
The causes of death of TEF and EMF groups.

Although all patients died within 11 months (median: two months) after EP, the total overall survival time was 1–106 months at the end of follow-up. The survival rates were 56.4%, 25.5%, 7.4% at one, three, and six months after EP, respectively. Furthermore, 38 patients died of hemorrhoea, 24 patients died of infection, 19 patients died of cancer cachexia, seven patients died of tumor metastasis, and six patients died of unknown causes. The one, three, and six months TAPs were 70.0%, 23.3%, and 6.7% for TEF and 50.0%, 26.6%, and 7.8% for EMF, respectively (*P* = 0.838). The causes of death are shown in **Figure 1**.

Seventy-seven patients developed EP after radiotherapy. The time from the beginning of radiotherapy to the definitive diagnosis of EP was 2–26 months, with a median time of six months. The survival analysis was performed using the time interval of three, six, and 12 months as the cut-off values and there was no statistical difference in the survival time after perforation. The one, three, and six months survival rates after EP were 62.2%, 28.9%, and 8.9% for patients with upper thoracic EC and 55.6%, 24.4%, and 6.7% for patients with middle thoracic EC, respectively. All patients with lower thoracic EC died within one month after EP and there was no significant difference in survival rates among the three groups (*P* = 0.068). The treatment after EP and the causes of death for the three groups are shown in **Table 2**.

**Table 2:** The treatment after EP and causes of death for patients with upper, middle and lower thoracic EC.

Location	Type of EP	Treatment	Death			
Upper thoracic(N=45)			Hemorrhage	Infection	Cancer cachexia	Others
	TEF(N=17)	Tube feeding(9)	2	2	4	1
		Stenting(6)	3	3	0	0
		Fluid infusion(2)	2	0	0	0
	EMF(N=28)	Tube feeding(16)	3	4	6	3
		Stenting(5)	5	0	0	0
		Fluid infusion(7)	6	1	0	0
Mid thoracic(N=45)	TEF(N=13)	Tube feeding(8)	3	3	2	0
		Stenting(5)	3	2	0	0
		Fluid infusion(0)	0	0	0	0
	EMF(N=32)	Tube feeding(14)	1	5	1	7
		Stenting(7)	4	1	0	2
		Fluid infusion(11)	4	2	5	0
Lower thoracic(N=4)	EMF(N=4)	Tube feeding(1)	0	0	1	0
		Stenting(3)	2	1	0	0
		Fluid infusion(0)	0	0	0	0

### Treatment and prognosis

**Figure 2:**
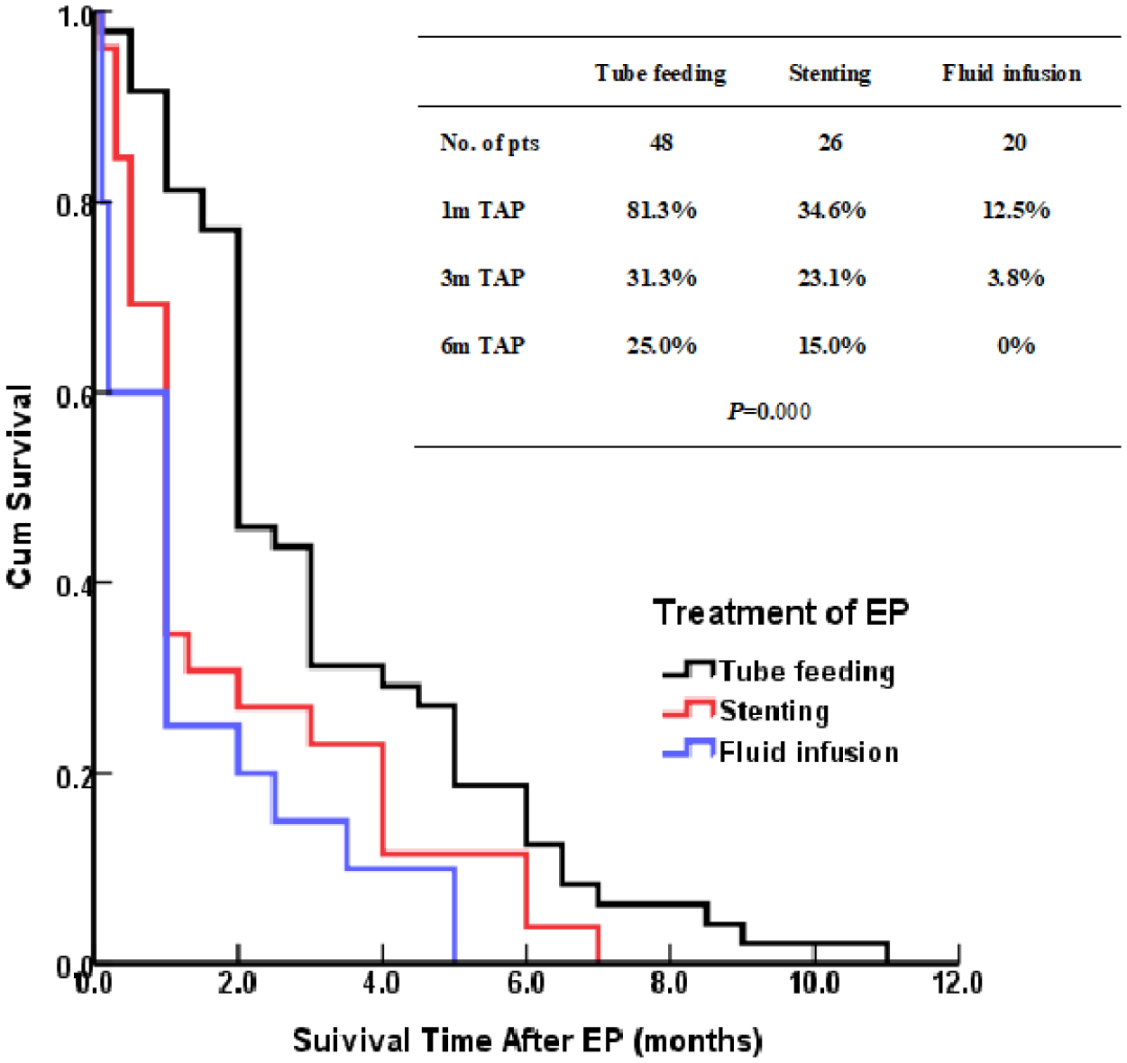
Kaplan–Meier curves for survival after EP.

**Figure 3:**
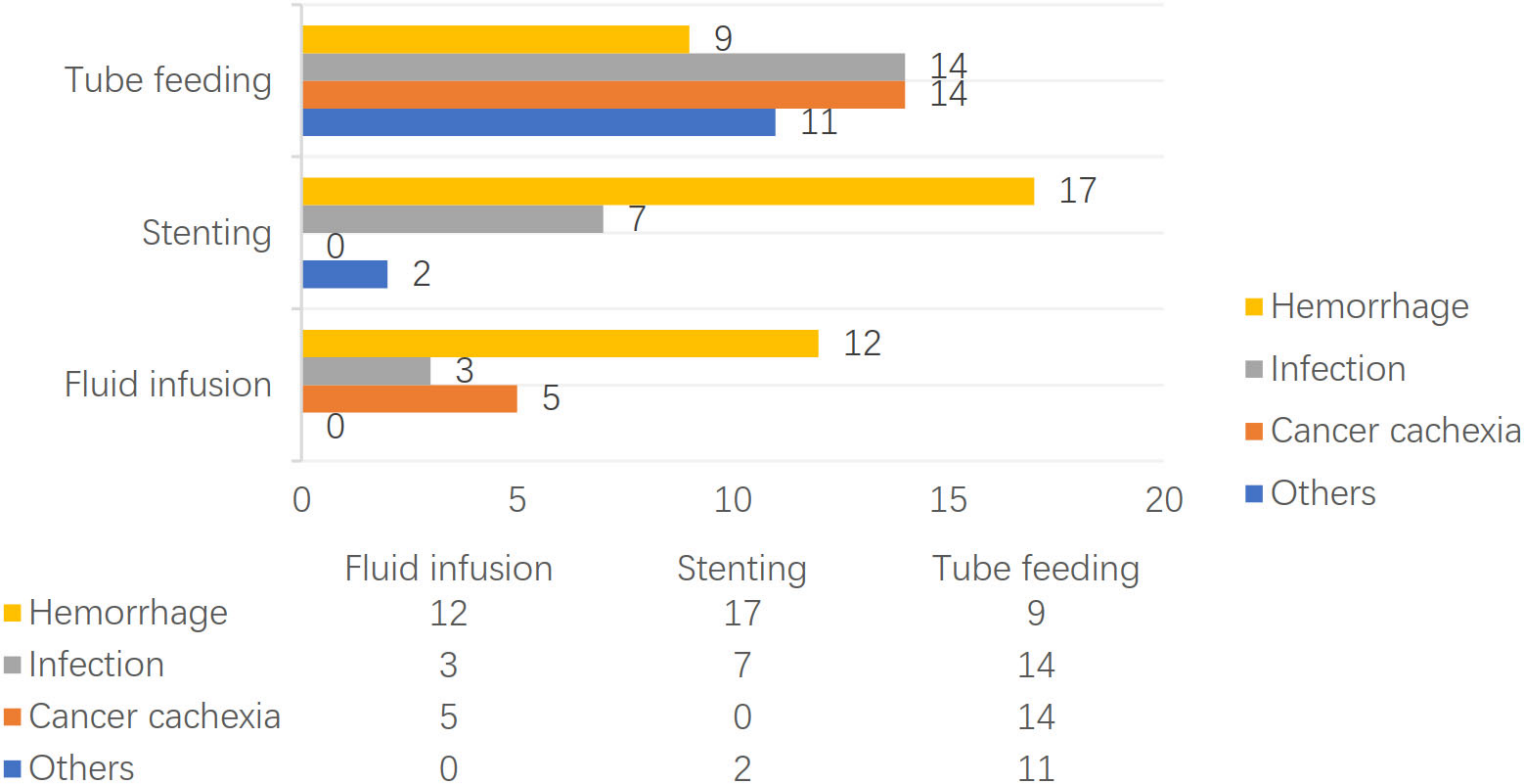
The treatment after EP and causes of death.

**Figure 4:**
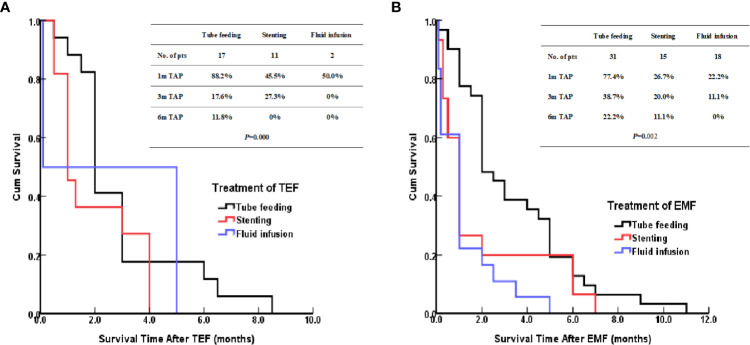
**(A)** Kaplan–Meier curves for survival after TEF. **(B)** Kaplan–Meier curves for survival after EMF.

**Figure 5:**
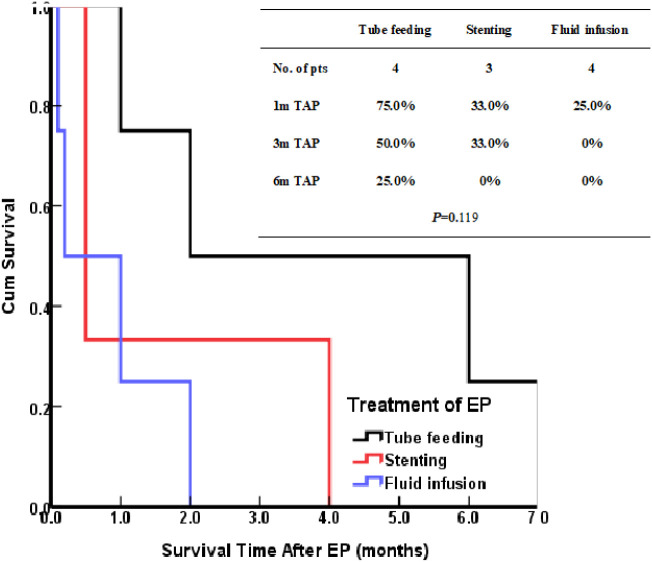
Kaplan–Meier curves for survival After EP of patients had EP during RT.

The patients treated by nasal feeding and gastrostomy were combined into the tube feeding group (48 cases) and their one, three, and six months survival rates after EP were 81.3%, 31.3%, and 12.5%, respectively. Compared with the other two treatment groups, the difference was statistically significant (*P <*0.001), as shown in **Figure 2**. The causes of death for the three treatment groups are shown in **Figure 3**. Patients with TEF and EMF were analyzed respectively. In the TEF group, the one, three, and six month survival rates after EP of patients treated by tube feeding (17 cases), esophageal stenting (11 cases), and fluid infusion (two cases) were 88.2%, 17.6%, and 11.8%; 45.5%, 27.3%, and 0%; and 50.0%, 50.0%, and 0% (*P* = 0.345), respectively, as shown in **Figure 4A**. In the EMF group, the one, three, and six months survival rates after EP of patients treated by tube feeding (31 cases), esophageal stenting (15 cases), and fluid infusion (18 cases) were 77.4%, 38.7%, and 12.9%; 26.7%, 20.0%, and 6.7%; and 22.2%, 11.1%, and 0% (*P*=0.002), respectively, as shown in **Figure 4B**. A total of 17 patients had EP during radiotherapy, six patients stopped radiotherapy, and 11 patients continued radiotherapy after treatment. Among the 11 patients who continued radiotherapy, the mean TAP was 2.2 (0.1–7.0) months. There was no significant difference in the survival rates after EP between the patients treated by tube feeding (four cases), esophageal stenting (three cases), and fluid infusion (four cases) (*P*=0.119), as shown in **Figure 5**.

## Discussions

Types of EP include TEF, EMF, esophagopleural fistula, and arterioesophageal fistula, among others; however, the most common of them are TEF and EMF. EP is a devastating and life-threatening complication. Of 277 patients, Xiao (15) reported that 62.2% and 81.5% of the patients died within three and six months after EP, respectively. In this study, all patients died within 11 months after EP, with a median TAP of two months. Moreover, nearly half (43.6%) of the patients died within one month after EP, while most of the patients died within six months after EP. The main complications in patients with EP are hemorrhage and infection (16), which are also the main causes of death among the patients. In this study, 66% of the 94 patients with EC died due to hemorrhage and infection. Particularly, 77% of the 30 patients with TEF died due to hemorrhage and infection; however, the proportion of death due to cancer cachexia, tumor metastasis, and other causes was relatively low. The main cause of death among the patients with EMF was hemorrhage (39.0%), whereas infection, cancer cachexia, tumor metastasis, and other causes account for 20% of the patients’ death.

Once EP occurs, there is almost no possibility of self-healing. With the development of the disease, the fistula becomes larger and aggravates the symptoms. If timely and effective treatment is not provided, the patient’s life is endangered. The treatment modalities are fistula closure, infection control, and adequate nutritional support. The methods of fistula closure include surgery, esophageal stenting, and tube feeding. Due to the poor constitution and tissue healing ability of patients with EP after radiotherapy, as well as the influence of radiotherapy on normal tissues and organs, surgical intervention, which is difficult in this scenario, can result in more postoperative complications (17). Therefore, the most common treatments are nasal feeding, gastrostomy, and esophageal stenting, which can ensure the intake of enteral nutrition. In addition, supportive therapy of intravenous infusion is also a treatment option. Comparative studies on the above-mentioned treatment methods are sparse. In this study, compared with the above-mentioned treatment methods for EP, it was found that the survival of patients treated by tube feeding, including gastrostomy and nasal feeding, was significantly better than the survival of patients treated by other methods. Moreover, these treatment methods can ensure the maximum intake of enteral nutrition and can maintain patients’ health. For patients undergoing esophageal stenting, although enteral nutrition can be guaranteed, the rate of death due to massive bleeding is significantly higher when compared to other causes. Moreover, previous studies have demonstrated that esophageal stents increase the probability of esophageal wall damage, perforation, and bleeding due to possible displacement and other factors. This may account for the shorter survival of the patients treated by esophageal stenting in this study. Consistent with the finding that tube feeding, including gastrostomy and nasal feeding, was significantly better than the other treatment methods, the good survival noted in this study was related to the maximum extent of ensuring adequate intake of enteral nutrition and maintaining the patients’ physique. Although enteral nutrition is also guaranteed for patients who underwent esophageal stenting, there were significantly more deaths due to hemorrhage than other causes. Previous investigations have demonstrated that the esophageal stent may increase the chances of esophageal wall injury, perforation, and hemorrhage due to possible displacement of the esophageal stent (18, 19), which may account for the short survival of the patients treated by esophageal stenting in this study.

Further analysis of the different treatment methods for EP showed that there was no statistical difference between patients with TEF treated by tube feeding, esophageal stenting, and fluid infusion; however, the survival of patients with EMF who were treated by tube feeding was significantly better than the survival of those treated by the other two methods (*P* = 0.002). For patients with different types of EP, the treatment options can be individualized. Patients with TEF present symptoms of dramatic cough, massive sputum, infection, and fever because the esophageal mucosa secretions or esophageal contents entered into the tracheal or bronchial from the fistula. An esophageal stent can close the fistula quickly and relieve the symptoms rapidly. Patients can opt for oral feeding, which not only ensures enteral nutrition support but also improves the quality of life (20, 21). For patients with TEF, esophageal stents can be used as appropriate; however, tube feeding is recommended for patients with EMF.

There was no statistical difference in the TAP between patients with TEF and EMF. There was no significant difference in the TAP between patients with upper, middle, and lower thoracic carcinoma. Seventy-seven cases of the EP occurred after radiotherapy. The patients were grouped according to the EP interval of three, six, and 12 months. There was no statistical difference in the survival rate between the three groups. For patients with EP after radiotherapy, the treatment modality is considered the most important prognostic factor affecting their survival.

Seventeen patients had EP during radiotherapy, six patients stopped radiotherapy, and 11 patients continued radiotherapy after treatment. Among the 11 patients, there was no statistical difference in the survival of those treated by tube feeding (4 cases), esophageal stenting (3 cases), and fluid infusion (4 cases); however, the survival of those treated by tube feeding was significantly better than the survival of those treated by other methods. The reason for the lack of statistical significance may be the small sample size. For patients with EP during radiotherapy, tube feeding during radiotherapy may be an effective treatment. However, given the limitation of the small number of cases in this study, further studies are needed in this regard.

The present study had several limitations. First, this study was a retrospective study with a long time span and prospective clinical study need be conduct to further investigate the patient’s clinical data and the process of symptom improvement. Second, due to the limited number of cases, surgical treatment was not included in this study. Third, the poor prognosis and the overall quality of life after perforation which can impact the outcome of various methods used to treat these perforations.

## Conclusion

Most patients with EP after radiotherapy died within six months, with low survival and poor prognosis. Tube feeding therapy can achieve relatively good survival, especially for patients with EMF. The survival of patients treated by tube feeding therapy is significantly better than the survival of those treated by other methods.

## Data availability statement

The raw data supporting the conclusions of this article will be made available by the authors, without undue reservation.

## Ethics statement

The studies involving human participants were reviewed and approved by Ethics Committee of Drug Clinical Trials, the Fourth Hospital of Hebei Medical University. The patients/participants provided their written informed consent to participate in this study.

## Author contributions

ZZ-G: Conceptualization, Methodology. ZC-J: Data curation, Writing- Original draft preparation. BW-W: Formal analysis. ZP: Writing - Review and Editing. SY-Z: Visualization. WY-J: Project administration. QX-Y: Supervision. All authors contributed to the article and approved the submitted version.
